# Factors associated with medication adherence to endocrine therapy in women with breast cancer: an analysis based on temporal self-regulation theory

**DOI:** 10.1007/s00520-026-10994-3

**Published:** 2026-07-13

**Authors:** Sheng-Miauh Huang, Ling-Ming Tseng, Chi-Cheng Huang, Han-Fang Cheng, Pei-Ju Lien

**Affiliations:** 1 College of Nursing, National Yang Ming Chiao Tung University, Taipei, Taiwan; 2 Department of Nursing, National Yang Ming Chiao Tung University Hospital, Yi-Lan, Taiwan; 3 Department of Surgery, Comprehensive Breast Health Center, Taipei Veterans General Hospital, Taipei, Taiwan; 4 College of Medicine, National Yang Ming Chiao Tung University, Taipei, Taiwan

**Keywords:** Breast cancer, Endocrine therapy, Temporal self-regulation theory, Medication adherence

## Abstract

**Purpose:**

Some patients with breast cancer show low adherence to endocrine therapy (ETs). The Temporal self-regulation theory (TST) can explain adherence to medication behavior. Using TST to demonstrate medication adherence in patients with cancer has not been previously investigated. This study aimed to describe the behavior of ET adherence in women with breast cancer and examine the factors affecting health behaviors by applying the TST model.

**Methods:**

A cross-sectional study design was used to collect data on connectedness beliefs, temporal valuations, intentions, self-regulatory capacity, behavioral prepotency, and ET adherence among 222 women with breast cancer.

**Results:**

Approximately one in seven women with breast cancer had low ET adherence. Under the guidance of the TST, it was found that factors directly influencing ET adherence included connectedness beliefs-barriers, intention, self-regulatory capacity, and behavioral prepotency. Both self-regulatory capacity and behavioral prepotency partially mediated the relationship between intention and medication adherence. Benefits and barriers to connectedness beliefs indirectly affected medication adherence through intentions. There were no significant moderators.

**Conclusions:**

This finding indicated that connectedness beliefs, intention, self-regulatory capacity, and behavioral prepotency are important factors of ET adherence. Future clinical interventions may target these specific psychological and behavioral mechanisms to effectively promote ET adherence in women with breast cancer.

## Introduction

Endocrine therapies (ETs), including tamoxifen and aromatase inhibitors, are effective treatments for improving prognosis in patients with hormone-positive breast cancer [1]. These proven ETs could be beneficial for reducing cancer recurrence and improving overall survival [2]. To achieve the desired therapeutic effect, ETs should be prescribed for 5–10 years, depending on the patient’s condition and risk of recurrence [1, 2]. The discontinuation rates of ETs from the 1-year to the 5-year mark were 5.38%, 16.70%, 32.27%, 51.52%, and 50.00% in China [3], while the rate of medicine adherence decreased from 94% at 1 year to 58% at 5 years in Italy [4]. These studies indicated that ET adherence at the fifth-year mark can fall below 60%. Poor ET adherence in breast cancer survivors often results in higher rates of cancer recurrence [5] and an increase in all-cause mortality [6, 7]. Thus, it is clear that ET adherence can be considered a necessary health behavior to be maintained or enhanced in patients with breast cancer.

Per previous studies, demographic and clinical characteristics influence medication adherence to ETs [4, 8, 9]. For example, patient age, employment status, and chronic disease status are important factors in explaining ET adherence [8, 9]. These findings can contribute to scientific knowledge but not as factors that can manipulate or intervene in health behaviors. Temporal self-regulation theory (TST) [10, 11] is a theoretical framework for explaining individual health behavior and could provide a direction for guiding behavior modification. It consists of a person-level motivational sphere and a situation-level momentary sphere. The person-level motivational sphere emphasizes cognitive processes through perceptions of the connection between current actions and future outcomes (connectedness beliefs) and the value placed on those outcomes (temporal valuations) that lead to behavioral intentions. Conversely, the situation-level momentary sphere focuses on the immediate situational influences of behavioral prepotency (the likelihood of a behavior occurring given habits and environmental cues) or self-regulatory capacity (the ability to exert top-down control over one's actions) that can override or influence intentions. Several scholars have investigated the utility of the TST to explain behavior and bridge the intention-behavior gap [12].

An evidence-based TST showed that medication adherence is proximally explained by three factors: intention, behavioral prepotency, and self-regulatory capacity [13]. These effect sizes explain health behaviors at weak to moderate levels [12]. Few studies have mentioned advanced moderating or mediating relationships in the TST [12, 13]. To date, no study has applied the TST to understand and analyze ET adherence in patients with breast cancer. This study aimed to describe the distribution of low adherence to ETs among Taiwanese women with breast cancer and to examine the factors affecting health behaviors using the TST model. Both moderation and mediation analyses were employed to investigate the roles of self-regulatory capacity and behavioral prepotency on the association between intention and medication adherence. The mediating role of intention in the person-level motivational sphere was also analyzed. These results are anticipated to offer valuable insights for breast cancer healthcare practitioners in developing tailored behavioral interventions and management strategies to optimize ET adherence.

## Materials and methods

### Study design and population

A cross-sectional research design was used to study adherence to ETs in a convenience sample of patients with breast cancer from a hospital in Taipei. Interviews were conducted with the women using a structured questionnaire from August 2024 to June 2025.

The sample size was estimated by modeling a previously published study on the TST in health and social behaviors [12]. Because the findings provide evidence for the TST to explain behavior with very weak-to-moderate effect sizes, the sample size was estimated to have an effect size of 0.2, an alpha value of 0.05, and a power value of 0.8. An initial estimate of at least 191 patients with breast cancer was required. The inclusion criteria were women aged 18 years or older who had been diagnosed with breast carcinoma, had received ET for at least 1 month, and were able to communicate in Chinese.

### Instruments

The study variables included patient characteristics (age, education level, marital status, and job status), clinical characteristics (cancer stage, medical history including diabetes and cardiovascular disease, and type of therapy), connectedness beliefs, temporal valuations, intentions, self-regulatory capacity, behavioral prepotency, and medication adherence. All data were collected from the outpatient department after the patients completed chemotherapy.

#### Connectedness beliefs

Connectedness belief in our study was measured by the questionnaire on Health Beliefs and Medication Adherence in Breast Cancer (HBMABC) [14]. The HBMABC scale comprises three properties: perceived susceptibility (three items), perceived benefits (three items), and perceived barriers (eight items). A Likert scale ranging from 1 to 5 was used to measure the level of agreement with each question (1 = totally disagree, 5 = totally agree). The scores on both the perceived susceptibility and perceived benefits subscales ranged from 3 to 15, whereas the scores on the perceived barriers subscale ranged from 8 to 40. A score of ≤ 8 (out of 15) on the perceived susceptibility subscale indicates low perceived susceptibility to cancer recurrence, a score of ≤ 11 (out of 15) on the perceived benefits subscale indicates low perceived benefit from ET, and a score of ≤ 12 (out of 40) on the perceived barriers subscale indicates low perceived barriers to ET.

#### Temporal valuation

The 5-trial adjusting delay task was designed to quickly assess an individual’s discount rate, with the flexibility to be used in various situations. Temporal valuation was assessed using a 5-trial Adjusting Delay discounting task [15]. The task was modified by Vaughn et al.’s study [16] in patients receiving adjuvant ET. Our respondents were asked on the first trial whether they would prefer half the amount per day (TWD 500,000) or the full amount (TWD 1,000,000) over 2 years. If the immediate option was selected, then the second trial shortened the delay to 4 days (e.g., TWD 500,000 in four hours or TWD 1,000,000 in four days). If the delayed option was selected in the first trial, then the second trial lengthened the delay (e.g., TWD 500,000 in 4 months or TWD 1,000,000 in 8 years). The delay for all subsequent tests was adjusted based on responses to the previous test. The task result represents the *k* parameter, which represents the discount rate and is often transformed using the natural logarithm (ln(k)). This transformation helps improve the normality of the data for statistical analysis and is a common practice when measuring the rate at which individuals discount future rewards.

#### Intention

The intention items were designed according to Ajzen, who argued that attitudes toward behavior, subjective norms, and the perception of behavioral control led to the formation of behavioral intention [17]. We designed the items of the Planned Behavior Questionnaire (PBQ) for medication according to the appendix in Fishbein and Ajzen’s publication [18]. Medication adherence was defined as taking medication daily at intervals and dosages prescribed for endocrine therapy. The questionnaire consisted of five questions, and a Likert scale ranging from 1 to 7 was used to measure the level of agreement for each question (1 = totally disagree, 7 = totally agree). The total medication adherence scores range from 5 to 35, with higher scores indicating better adherence.

#### Self-regulatory capacity

Self-regulatory capacity in our study was measured with the 13-item Brief Self-Control Scale (BSCS) [19]. Items were answered on a 5-point Likert scale ranging from 1 (not at all like me) to 5 (very much like me). The total possible BSCS score ranges from 13 to 65, with higher scores indicating higher levels of self-control.

#### Behavioral prepotency

This study defines behavioral prepotency as a prioritized response to health behaviors such as automaticity. The self-reported behavioral automaticity index (SRBAI) [20], consisting of a four-item scale, was used in our study to measure behavioral prepotency. Participants were asked to rate their agreement with each statement on a scale ranging from 1 to 7. The possible total SRBAI score ranges from 4 to 28; the higher the score, the better the automaticity level.

#### Medication adherence

The Morisky Medication Adherence Scale-8 (MMAS-8) [21], comprising eight items, was used to assess medication adherence in patients with breast cancer. Lin et al. conducted a validity and reliability study using the Taiwanese version [22]. Each of the first seven items had two possible responses (yes/no), while the 8th item was answered on a 5-point Likert scale. The possible total medication adherence score ranges between 0 and 8; the higher the score, the better the adherence level. A total score < 6 indicated low adherence.

### Statistical analysis

All data were analyzed using the IBM Statistical Package for the Social Sciences for Windows (SPSS, version 26.0; IBM Corp., Armonk, NY, USA) and the R Integration Package [23]. Descriptive data are presented as percentages, means, and standard deviations. T-tests and analysis of variance were used to examine differences between groups. Binary logistic regression analysis was used to identify variables associated with medication adherence. Model 2 of the moderation analysis within SPSS and R codes can be used to examine the roles of two moderators on the relationship between an independent variable and a dependent variable [23]. Model 4 of mediation analysis with 5000 bootstrap samples and 95% bias-corrected bootstrap intervals for all indirect variables was performed to investigate whether the relationship between intention and medication adherence was mediated by self-regulatory capacity or behavioral prepotency and whether the relationship between connectedness beliefs and medication adherence was mediated by intention [23].

## Results

### Sociodemographic and clinical characteristics

Table 1 presents the demographic and clinical characteristics of the study participants. In total, 280 women met the inclusion criteria. Of these, 58 refused to participate because they were tired or uninterested. The response rate was 79.3%. Overall, 222 participants were enrolled in this study. The respondents ranged in age from 27 to 80 years (56.3 ± 8.95 years), and 68.5% were married. Table 1 shows the demographic and clinical characteristics of the patients. Most were college graduates (66.7%), had full-time jobs (57.7%), and some had diabetes (7.2%) or cardiovascular disease (17.1%). Regarding clinical data, approximately 94.1% of the patients had undergone surgery, and more than half had received chemotherapy or radiotherapy (Table 1). Low medication adherence was observed in approximately 14.4% of patients.

**Table 1:** Differences in demographic and health related data between low and high medication adherence in breast cancer women receiving endocrine therapy (*n =* 222)

Variable	All		MMAS-8					
			Low adherence (score ≥ 6) (*n* = 32, 14.4%)			High adherence (score < 6) (*n* = 190, 85.6%)		
	*n*	%	*n*		(%)	*n*	(%)	*χ*^2^/t
Age (years)								1.50
25–50	61	27.5		11	34.4	50	26.3	
51–65	126	56.8		15	46.9	111	58.4	
≥ 66	35	15.8		6	18.7	29	15.3	
Education								1.04
Elementary school or junior high school	16	7.2		1	3.1	15	7.9	
Senior high school	58	26.1		8	25.0	50	26.3	
College at least	148	66.7		23	71.9	125	65.8	
Marital status								2.60
Single	47	21.2		10	31.3	37	19.5	
Married	152	68.5		20	62.5	132	69.5	
Divorced or widowed	23	10.4		2	6.3	21	11.1	
Currently working								0.03
Yes	128	57.7		18	56.3	110	57.9	
No	94	42.3		14	43.8	80	42.1	
Diabetes								0.26
Yes	16	7.2		3	9.4	13	6.8	
No	206	92.8		29	90.6	177	93.2	
Cardiovascular disease								0.17
Yes	38	17.1		6	81.3	32	16.8	
No	184	82.9		26	18.8	158	83.2	
Cancer stage								0.07
Ⅰ and Ⅱ	191	86.0		28	87.5	163	85.8	
Ⅲ and Ⅳ	31	14.0		4	12.5	27	14.2	
Cancer location								0.61
Right	99	44.6		15	46.9	84	44.2	
Left	108	48.6		14	43.8	94	49.5	
Bilateral	15	6.8		3	9.4	12	6.3	
Surgery								0.51
Yes	209	94.1		31	96.9	178	93.7	
No	13	5.9		1	3.1	12	6.3	
Chemotherapy								0.96
Yes	121	54.5		20	62.5	101	53.2	
No	101	45.5		12	37.5	89	46.8	
Radiotherapy								2.58
Yes	126	56.8		14	43.8	112	58.9	
No	96	43.2		18	56.3	78	41.1	
HBMABC—susceptibility								0.51
Low	164	73.9		22	68.8	142	74.7	
High	58	26.1		10	31.3	48	25.3	
HBMABC -benefit								6.42*
Low	128	57.7		25	78.1	103	54.2	
High	94	42.3		7	21.9	87	45.8	
HBMABC -barrier								29.21**
Low	148	66.7		8	25.0	140	73.7	
High	74	33.3		24	75.0	50	26.3	
ED 50	2203.8	2734.4		1940.1	2607.2	2248.2	2759.3	−0.59
PBQ	31.4	6.0		24.6	8.6	32.5	4.5	−5.06**
SRBAI	23.7	5.8		17.3	7.2	24.8	4.8	−5.71**
BSCS	49.4	5.9		45.9	7.4	50.0	5.4	−3.70**

The chi-squared test or independent t-test was used to examine the significance of differences in characteristics between women with low or high medication adherence; *the significance level was set at *p* < 0.05; **p < 0.01

*BSCS* Brief Self-Control Scale, *ED* effective delay, *HBMABC* Health Beliefs and Medication Adherence in Breast Cancer, *PBQ* Planned Behavior Questionnaire for Medication, *SRBAI-M* Self-Report Behavioral Automaticity Index for Medication

### Result of TST constructs on medication adherence

Based on TST self-regulation theory (TST theory), it was hypothesized that an individual’s intention, self-regulatory capacity, and behavioral prepotency would affect medication adherence behavior. Therefore, an independent samples t-test was first used to analyze the hypothesis, which revealed a significant difference in scores for intention, self-regulatory capacity, and behavioral prepotency between those with low medication adherence and those with high medication compliance (Table 1). Then, binary logistic regression analysis showed that those with higher intention scores had a higher chance of being high medication adherents compared to those with low intention scores (PBQ: OR = 1.11, 95% CI 1.04 to 1.20, *p* < 0.01, Table 2), and those with higher behavioral prepotency scores had a higher chance of being high medication adherents compared to those with low behavioral prepotency scores (SRBAI: OR = 1.11, 95% CI 1.03 to 1.20, *p* = 0.01, Table 2). Those with higher self-regulatory capacity scores had a higher chance of being high medication adherents than those with low self-regulatory capacity scores (BSCS: OR = 1.10, 95% CI 1.01 to 1.18, *p* = 0.02, Table 2).

**Table 2:** Binary logistic regression model results on medication adherence in patients with breast cancer receiving endocrine therapy (*n =* 222)

Variable	*B*	SE	*P*	Exp(B)	95% CI for EXP(B)	
					Lower	Upper
PBQ	0.11	0.04	< 0.01	1.11	1.04	1.20
SRBAI	0.11	0.04	0.01	1.11	1.03	1.20
BSCS	0.09	0.04	0.02	1.10	1.01	1.18
Constant	−8.13	2.11	< 0.01	< 0.01		

*BSCS* Brief Self-Control Scale, *PBQ*, Planned Behavior Questionnaire for Medication; *SRBAI*, Self-Report Behavioral Automaticity Index for Medication

### Results of moderation and mediation analysis to explain medication adherence

**Fig. 1:**
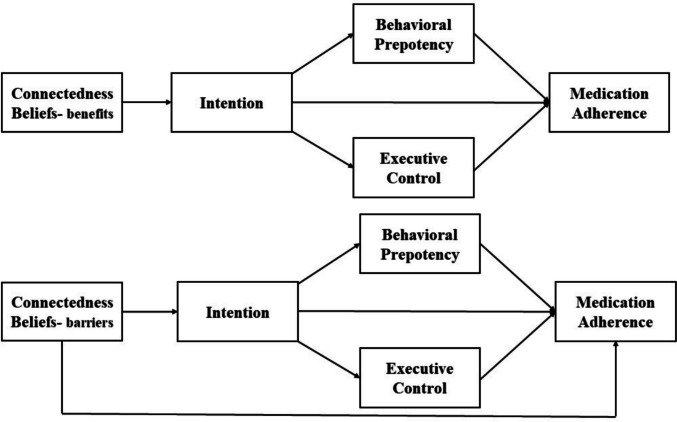
Explanatory model of medication adherence in breast cancer women receiving endocrine therapy

The mediation analysis was conducted using model 4, and the final results showed that intention had a direct effect on medication adherence and that either self-regulatory capacity or behavioral prepotency was a partial mediator of the relationship between intention and medication adherence (BSCS: indirect effect = 0.018, 95% CI 0.002 to 0.045; SRBAI: indirect effect = 0.073, 95% CI 0.027 to 0.134, Table 3), and intention was a partial mediator of the relationship between barriers of connectedness beliefs and medication adherence (indirect effect = −0.595, 95% CI −1.057 to −0.287, Table 3). Additionally, the intention was a complete mediator on the relationship between the benefits of connectedness beliefs and medication adherence (direct effect = 0.710, 95% CI −0.314 to 1.735; indirect effect = 0.465, 95% CI 0.209 to 0.838, Table 3). The explanatory model of medication adherence in women with breast cancer receiving endocrine therapy is shown in Fig. 1.

**Table 3:** Mediation models on medication adherence in breast cancer women receiving endocrine therapy (*n =* 222)

X variable	Mediation variable		Effect	95% CI
PBQ	BSCS			
		Direct effect	0.163	0.101 to 0.226
		Indirect effect	0.018	0.002 to 0.045^✝^
PBQ	SRBAI			
		Direct effect	0.111	0.038 to 0.184
		Indirect effect	0.073	0.027 to 0.134^✝^
HBMABC -Benefit	PBQ			
		Direct effect	0.710	−0.314 to 1.735
		Indirect effect	0.465	0.209 to 0.838^✝^
HBMABC -Barrier	PBQ			
		Direct effect	−1.684	−2.618 to −0.749
		Indirect effect	−0.595	−1.057 to −0.287^✝^

*BSCS* Brief Self-Control Scale, *HBMABC* Health Beliefs and Medication Adherence in Breast Cancer, *PBQ* Planned Behavior Questionnaire for Medication, *SRBAI* Self-Report Behavioral Automaticity Index for Medication

^✝^Bootstrap confidence interval

Total effect model not available with dichotomous Y; effect size option not available with dichotomous Y; direct and indirect effects of X on Y are on a log-odds metric

Moderating effect analysis was conducted using Model 2 within SPSS and R codes, and the final results showed that there was no moderating effect on the relationship between PBQ and medication adherence, regardless of the BSCS or SRBAI variables.

## Discussion

The current study found that approximately 14.4% of the patients undergoing ETs in Taiwan had low medication adherence. A Japanese study showed that the rate of low medication adherence was about 15% [24] and a report from Turkish scholars showed that it was 16% [8]. While these figures are similar, direct comparisons require caution due to the use of different assessment instruments. Intriguingly, these prevalence rates are lower than those in Asians with chronic diseases (e.g., 48% for hypertension) [25]. This may be attributed to perceived beliefs about the severity and threat of breast cancer [26].

According to the TST [10, 11], connectedness beliefs refer to the degree to which individuals believe their current actions will influence future outcomes. This implies that patients with breast cancer are convinced that ETs can be effective in treating cancer and prolonging life. Our results indicated that the benefits of connectedness beliefs had an effect on completing medication through intention on ET adherence, while the barriers to connectedness beliefs had both direct and indirect impacts on ET adherence through intention. In contrast to the TST, there was a possible direct effect on the relationship between connectedness beliefs (especially barriers) and medication adherence. This finding may help theorists rethink the value of beliefs and their relationship with behavior. We also suggest that clinical practice should target belief education to enhance benefits and minimize barriers to improving ET adherence in patients with breast cancer.

Research related to the TST has conclusively found that factors associated with healthy behaviors include intention, behavioral prepotency, and self-regulatory capacity [12, 27]. However, there is a lack of evidence on the moderating effect of behavioral prepotency and self-regulatory capacity on the relationship between intentions and healthy behavior [12]. In agreement with previous findings, our results indicated that these constructs are important factors in explaining ETs adherence. Our analysis also provides evidence that behavioral prepotency and self-regulatory capacity do not have a moderating effect on the relationship between intentions and ET adherence. Furthermore, we found a mediating effect of behavioral prepotency and self-regulatory capacity on the relationship between intentions and adherence to ET. Our findings present a new perspective on the relationship between the three constructs and ET adherence behavior within the TST theory; however, further research is needed to validate this. Several studies have been conducted to improve medication adherence by using strategies similar to those of the TST concept [28–31]. Future studies should simultaneously investigate these three constructs and target them for behavioral interventions to promote medication intention, self-control, and habits in this population.

Based on the TST, temporal valuation means that outcomes perceived as closer in time are usually of higher value. Our results showed that temporal evaluation was not significantly associated with adherence to ET. This was consistent with research regarding the delayed behavior of breast cancer diagnosis [32] but inconsistent with a study on body weight control [33]. The use of different instruments to measure prescribing behavior may be one reason for these different results [16]. Whether particular personality trait differences affect time perception requires further investigation [33].

### Strengths and limitations

The study uses the TST to design, collect, and analyze five constructs: connectedness beliefs, temporal valuations, intention, self-regulatory capacity, and behavioral prepotency. To the best of our knowledge, this study is the first to validate the TST for medication adherence in patients with cancer. All instruments were reliable and trustworthy. This study had some limitations. First, there may have been a selection bias in our study because the participants were from only one hospital in Taipei. Therefore, the number of those who attended other hospitals may not have been the same as those who participated in the survey. Future studies should recruit a larger sample of hospitals in different districts and at different levels. Second, the findings were derived from the analysis of a cross-sectional study. Whether the changes in ET adherence measured in longitudinal studies are consistent with the current conclusions requires further investigation. Third, ET adherence was measured based on an individual’s own perceptions in our study. This may have resulted in an overestimation of ET adherence. Whether these results are consistent with the actual state of ETs use by patients with cancer warrants further study. Fourth, we did not collect specific data on the start time of ET and the ET regimens for each patient, so we could not determine the annual rate of ET adherence and the impact of different ET regimens on it. Finally, while this study effectively utilized TST to elucidate the psychological factors of ET adherence, it inherently overlooked physiological covariates. The potentially confounding or direct impact of ET-related symptoms and side effects on non-adherence was not comprehensively examined.

## Conclusion

Approximately one-seventh of patients with breast cancer had low ET adherence in Taiwan. Among the five constructs based on the self-regulation theory (TST) used to explain ET adherence, connectedness beliefs, intention, self-regulatory capacity, and behavioral prepotency were essential factors. Furthermore, a mediating effect may be present in the TST rather than a modulating effect. Intentions play a vital role in both the motivational and momentary spheres of the TST. Future interventions should be grounded in these important constructs of the TST, focusing on optimizing beliefs, intentions, self-control, and habits while also considering the individual characteristics of the breast cancer population.

## Data Availability

The data that support the findings of this study are not publicly available due to participant confidentiality but are available from the corresponding author upon reasonable request.
